# Enhanced Wavelet-Based Medical Image Denoising with Bayesian-Optimized Bilateral Filtering

**DOI:** 10.3390/s24216849

**Published:** 2024-10-25

**Authors:** Mehdi Taassori

**Affiliations:** Institute of Cyberphysical Systems, John von Neumann Faculty of Informatics, Obuda University, 1034 Budapest, Hungary; taassori.mehdi@uni-obuda.hu

**Keywords:** wavelet, bilateral filter, Bayesian optimization, denoising, medical image

## Abstract

Medical image denoising is essential for improving the clarity and accuracy of diagnostic images. In this paper, we present an enhanced wavelet-based method for medical image denoising, aiming to effectively remove noise while preserving critical image details. After applying wavelet denoising, a bilateral filter is utilized as a post-processing step to further enhance image quality by reducing noise while maintaining edge sharpness. The bilateral filter’s effectiveness heavily depends on its parameters, which must be carefully optimized. To achieve this, we employ Bayesian optimization, a powerful technique that efficiently identifies the optimal filter parameters, ensuring the best balance between noise reduction and detail preservation. The experimental results demonstrate a significant improvement in image denoising performance, validating the effectiveness of our approach.

## 1. Introduction

Medical image denoising is a crucial process in the field of medical imaging, as it directly impacts the quality and reliability of diagnostic images. High-quality images are vital for accurate diagnosis and treatment planning, but medical images often suffer from noise introduced during the acquisition process. This noise can obscure important details, making it challenging for healthcare professionals to detect and analyze abnormalities. Therefore, effective denoising techniques are essential to enhance image clarity, preserve critical features, and ultimately improve diagnostic accuracy.

Over the years, various denoising methods have been developed, each with their strengths and limitations. Traditional filtering methods, such as median [1], non-local means [2,3,4], bilateral filters [5,6,7,8,9,10,11,12], wavelets [13,14,15,16,17,18], and Kalman filter [19,20], have been widely used due to their simplicity and effectiveness in smoothing noise while maintaining edge information. Transform domain methods, including wavelet transforms and Fourier transforms, focus on manipulating the image in a different domain to isolate and reduce noise components.

The bilateral filter is a widely used technique in image processing, and it is known for its ability to perform edge-preserving smoothing while reducing noise. Its unique approach lies in combining both spatial and intensity information to selectively smooth an image, which helps maintain important features like edges. Given its effectiveness, the bilateral filter has been the foundation for numerous advancements in various image processing applications. In this context, several studies have explored, leading to innovations that address specific challenges, such as noise reduction. In [5], the authors introduce the adaptive bilateral filter (ABF), a technique designed for sharpness enhancement and noise removal in images. The ABF distinguishes itself by enhancing edge sharpness through slope modification, avoiding the overshoot or undershoot artifacts typically associated with traditional methods, like the unsharp mask. Unlike previous slope restoration techniques, the ABF does not require edge detection or orientation analysis. Instead, it utilizes a range filter with adaptive offset and width to transform the image’s histogram, effectively smoothing noise while enhancing edges and textures. The study in [6] presents a supervised learning procedure aimed at estimating the relationship between local image features and the optimal parameters of an adaptive bilateral filter. Specifically, two entropy-based features are utilized to characterize the properties of the image at a local scale. In [7], the authors employed the bilateral filter (BF) as an edge-preserving method for removing Rician noise in MR images. Recognizing that the performance of denoising heavily depends on the choice of BF parameters, they proposed a novel approach by optimizing these parameters using a genetic algorithm (GA). In their study, Rician noise with varying variances was added to simulated T1-weighted brain MR images. The GA was then applied to these noisy images to determine the optimal filter parameters, resulting in improved noise removal performance. In [9], the authors extend the classical bilateral filter by introducing an adaptive bilateral filter, where the center and width of the Gaussian range kernel vary from pixel to pixel. While the traditional bilateral filter uses a fixed Gaussian range kernel along with a spatial kernel for edge-preserving smoothing, this adaptive version allows for enhanced flexibility, making it suitable not only for sharpening and noise removal but also for artifact removal and texture filtering. The authors propose a fast algorithm for adaptive bilateral filtering that significantly reduces computational complexity, particularly as it does not scale with the spatial filter width. Their approach leverages the observation that filtering can be conducted purely in range space using a locally defined histogram. By approximating the histogram with a polynomial and replacing the finite range-space sum with an integral, the adaptive filter is efficiently approximated using analytic functions. In [10], the authors present a fast implementation of bilateral filtering that leverages an optimal expansion of the filter kernel into a sum of factorized terms. These terms are determined by minimizing the expansion error using a mean square error approach, resulting in a solution that involves the eigenvectors of a square matrix. This method simplifies the bilateral filtering process to the computation of a few Gaussian convolutions, for which efficient algorithms are widely available. Additionally, the expansion functions are optimized for the histogram of the input image, enhancing accuracy. The research in [11] addresses the limitations of existing fast algorithms for bilateral and non-local means filtering, which are typically confined to grayscale images and struggle with high-dimensional data like color or hyperspectral images, patch-based data, and flow fields. To overcome these challenges, the authors introduce a fast algorithm specifically designed for high-dimensional bilateral and non-local means filtering. Unlike traditional methods that approximate the data or filter kernel, their approach locally approximates the kernel using weighted and shifted copies of a Gaussian, with the weights and shifts derived from the data itself. Recent advances in fast implementations of bilateral and non-local filters have focused on approaches using lattice and vector quantization techniques, such as clustering in higher dimensions. Despite these developments, these methods often face inefficiencies due to the complexities associated with resampling processes and filtering high-dimensional resampled signals. To address these challenges, [12] introduces a novel approach that simplifies the process by employing scalar resampling of the high-dimensional signal after decorrelation. This approach enables the use of multi-rate signal processing techniques, enhancing both efficiency and accuracy. The proposed Gaussian lifting framework takes advantage of the similarities between separable wavelet transforms and Gaussian pyramids, providing an effective solution for bilateral and non-local means filtering in high-dimensional contexts.

Wavelet transforms have become an essential tool in image processing, offering a powerful framework for analyzing and processing images across multiple scales. This capability is exemplified in the MSWSR model [21], which utilizes multi-level wavelet coefficients for multi-scale super resolution. By employing a CNN for low-frequency wavelet prediction and an RNN for high-frequency components, MSWSR captures detailed multi-scale information. Additionally, a side-window convolution kernel reduces model complexity, showcasing the efficiency of wavelet-based approaches in enhancing image reconstruction. In this context, recent advancements in deep learning super-resolution models, particularly the WMRSR framework [22], address limitations by capturing auxiliary information across multiple subspaces. These models emphasize the importance of exploring correlations between wavelet and spatial domain features, which are often overlooked. The WMRSR generates wavelet multi-resolution inputs by combining wavelet sub-bands and spatial content, effectively utilizing features from both domains. By adaptively fusing these features, the WMRSR ensures a comprehensive representation that enhances reconstruction quality. Wavelets provide both time and frequency localization, making them particularly effective for tasks like denoising and compression. Their ability to decompose an image into different frequency components allows for targeted manipulation of various image details, from fine textures to broader structures. Due to their flexibility and effectiveness, wavelets have been extensively studied and adapted in various image processing applications. Numerous studies have proposed novel wavelet-based techniques that enhance image quality and improve processing efficiency, addressing challenges such as noise reduction. In [13], the authors propose an adaptive, data-driven threshold for image denoising using wavelet soft thresholding. This threshold is derived within a Bayesian framework, with the generalized Gaussian distribution (GGD) serving as the prior for the wavelet coefficients. Additionally, the paper investigates and validates that lossy compression can be effectively utilized as a denoising technique. In [14], the authors propose a spatially adaptive wavelet thresholding method for noise removal, building on the well-established effectiveness and simplicity of wavelet thresholding. The method leverages context modeling to adapt the denoising process to varying image characteristics. Each wavelet coefficient is treated as a random variable following a generalized Gaussian distribution with an unknown parameter. Context modeling is then employed to estimate this parameter for each coefficient, allowing the thresholding strategy to be dynamically adjusted. In [15], the authors present a wavelet-based method for noise reduction in CT images that preserves structural details. The technique is designed to work with various CT reconstruction methods and operates on the assumption that image data can be decomposed into meaningful information and temporally uncorrelated noise. By generating two spatially identical but independently noisy images, the method analyzes the correlations between the wavelet representations of these images. Wavelet coefficients with low correlation, indicative of noise, are suppressed, while those with high correlation, representing structures, are preserved. The final noise-reduced image is reconstructed from the averaged and weighted wavelet coefficients of the input images. The authors in [16] investigate the application of encoding–decoding CNNs for noise reduction. They build on the theory of deep convolutional frameworks, which connects signal processing principles with CNN architectures. Unlike traditional CNNs, which often fail to provide the necessary redundancy for perfect reconstruction, this paper focuses on CNN architectures that meet the conditions for achieving perfect reconstruction. This investigation leads to the development of the learned wavelet-frame shrinkage network. The work presented in [17] introduces a wavelet-inspired invertible network that combines the benefits of wavelet-based and learning-based methods. The wavelet-inspired invertible network comprises K-scale lifting-inspired invertible neural networks, sparsity-driven denoising networks, and a noise estimation network. Lifting-inspired neural networks, which are inspired by the lifting scheme used in wavelets, are employed to learn a non-linear redundant transform with perfect reconstruction properties, enhancing noise removal. The denoising network applies a sparse coding method, and the noise estimation network determines the noise level from the input image to adaptively adjust the soft thresholds in lifting-inspired neural networks. The forward transform of lifting-inspired neural networks generates a redundant multi-scale representation, and the final denoised image is reconstructed using the inverse transform of lifting-inspired neural networks with the denoised and the original channel. The challenge of obtaining high-quality training datasets for medical image reconstruction is significant, especially due to noise and artifacts in CT and MRI, which make it difficult to accurately estimate data distribution gradients. This issue reduces the performance of score-based generative models in such applications. To address this problem, the researchers in [18] proposed a wavelet-improved denoising technique that works with score-based generative models to ensure more reliable and stable training. Their method integrates a wavelet sub-network with the standard score-based generative model sub-network into a unified framework, correcting inaccurate data distribution gradients and enhancing overall stability. This approach allows the network to learn accurate scores even when handling noisy samples, leading to more precise and dependable reconstructed images.

Researchers have often combined different methods to take advantage of each, creating more powerful and effective solutions. This approach helps to overcome the limitations of individual methods, leading to better performance in various applications. The benefits gained through these combinations have proven to be a valuable strategy. Researchers in [20] present a novel hybrid approach to enhance the denoising process for medical images. They start using an adaptive Kalman filter that adjusts its parameters based on the noise characteristics of the image, providing a more accurate estimation of the true image state. After this initial noise reduction, a non-local means method and a median filter are applied to further refine the image. While the non-local means method reduces noise by comparing similar image patches and the median filter eliminates residual noise, their effectiveness depends on optimal parameter settings, which traditionally require extensive computation. To address this, the researchers introduce Latin square optimization, an efficient method for selecting the best parameters for non-local means.

Hyperparameter tuning is crucial for optimizing model performance, especially when dealing with large datasets, where manual tuning is impractical. Bayesian optimization offers an efficient and systematic way to fine tune these settings, leading to enhanced accuracy. The researchers in [23] addressed this challenge by applying the Bayesian hyperparameter optimization algorithm to enhance model performance. Bayesian optimization, suitable for tuning any noisy black box function, was effectively used in this study to determine the optimal hyperparameter values. This approach not only saved time but also significantly improved the overall performance of the model.

In this paper, we utilized wavelet techniques for denoising, which are highly effective in preserving image details while reducing noise. Following this, we applied bilateral filtering as a post-processing step to further enhance the image by smoothing while maintaining edge sharpness. The combination of these two approaches leverages the strengths of both methods, providing a more robust solution for image enhancement. A crucial aspect of our approach is finding the optimal parameters for the bilateral filter to ensure the best performance. To achieve this, we employed Bayesian optimization, a powerful method that systematically explores the parameter space, efficiently identifying the most effective settings to maximize the performance of the bilateral filter.

## 2. Motivation

Combining different image denoising filters can provide significant advantages over using a single filter, particularly in enhancing denoising performance and preserving critical image details. One key benefit of combining filters is the ability to leverage the strengths of each individual filter. Different filters are designed to target specific types of noise or image features, and combining them allows for a more comprehensive approach to noise reduction. For instance, one filter might be more effective at removing high-frequency noise, while another might excel in preserving edges and fine details. By integrating these filters, it is possible to achieve a balance between noise suppression and detail preservation, leading to superior image quality. Another advantage of combining filters is the ability to adapt to varying noise levels and patterns across different images or regions within an image. Filters that are effective for one type of noise may not perform as well on another. A combined approach can dynamically adjust to different noise characteristics, providing a more robust and versatile solution. Moreover, combining filters can help to mitigate the limitations or weaknesses of individual filters, resulting in fewer artifacts and better overall performance. In [20], the authors demonstrated the effectiveness of combining filters by integrating an adaptive Kalman filter with non-local means (NLM) and median filters. This combination yielded better denoising results than using any of the individual filters alone, highlighting the potential for combined filtering techniques to enhance image quality in diverse scenarios.

Wavelet filters offer several advantages for image denoising, primarily due to their ability to perform multi-resolution analysis. This capability allows wavelet filters to capture both coarse and fine details, making them effective in targeting and reducing noise across different frequency components of an image. Wavelet filters are also efficient in noise reduction through the use of thresholding techniques, which can selectively suppress noise without significantly affecting important image structures. Furthermore, the scalability of wavelet-based denoising makes it adaptable to images with varying levels of detail and complexity. However, wavelet filters can sometimes introduce artifacts, such as ringing effects. They may also struggle with preserving sharp edges and small details in areas of high contrast, which can result in a loss of fine structural information. On the other hand, bilateral filters are well known for their ability to preserve edges while reducing noise. By combining spatial and intensity information, bilateral filtering effectively maintains sharp edges and intricate details, offering a balance between noise reduction and detail preservation. The simplicity and effectiveness of bilateral filters make them widely applicable for various image processing tasks, and their non-linear nature makes them robust against different types of noise, ensuring that images retain a natural appearance. However, bilateral filters can be computationally expensive, particularly for large images or when using a large filter size, as each pixel must be processed based on its neighborhood. Combining bilateral filtering with wavelet filtering can help address this issue. By applying wavelet filtering first, the image is broken down into different frequency components and noise levels. This preprocessing step reduces the complexity of the bilateral filter by simplifying the image structure, allowing it to operate more efficiently. As a result, the combination can significantly reduce the computational load and improve overall performance.

Combining wavelet and bilateral filters leverages the strengths of both techniques, resulting in more effective image denoising while mitigating their individual limitations. The wavelet filter’s ability to perform multi-resolution analysis complements the bilateral filter’s edge-preserving capabilities. This combination allows for efficient noise reduction across various frequency bands while maintaining sharp edges and intricate details. By first using wavelet decomposition to target different noise levels and then applying bilateral filtering for localized edge preservation, the combined approach achieves improved denoising performance. The combined use of wavelet and bilateral filters also reduces the risk of artifacts typically associated with aggressive wavelet thresholding, as the bilateral filter’s edge-aware smoothing can refine the denoised image. Additionally, this combination can be more robust to varying noise characteristics, offering a more adaptable solution that performs well under different noise conditions. Overall, the integration of wavelet and bilateral filtering methods enhances image quality, making it highly effective for applications requiring both noise suppression and detail preservation.

Additionally, the performance of bilateral filters is highly dependent on the careful tuning of parameters, such as filter size and sigma values, which can vary with different images and noise levels. Improper parameter settings may lead to either oversmoothing or inadequate noise removal. Complex systems often have numerous tunable configuration parameters, which are typically set and hard coded by different developers or teams. Optimizing these parameters jointly can lead to substantial performance improvements. Bayesian optimization has become a powerful method for this task, offering a systematic approach to automate the optimization process, enhance product quality, and increase overall productivity [24]. In this paper, Bayesian optimization was employed to efficiently find the optimal parameters for the bilateral filter. This approach leverages probabilistic models to explore the parameter space systematically, ensuring that the chosen parameters provide the best balance between noise reduction and detail preservation for various images and noise conditions. Bayesian optimization was chosen for parameter tuning in this study due to its efficiency and effectiveness in optimizing complex, high-dimensional spaces. Unlike grid search or random search, which can be computationally expensive and inefficient, Bayesian optimization uses probabilistic models to guide the search for optimal parameters. It intelligently explores the parameter space by balancing the exploration of new areas and the exploitation of known good areas, which leads to fewer evaluations needed to find the best parameters. This is particularly advantageous when dealing with computationally intensive tasks, like bilateral filtering, where each evaluation involves significant processing. Using Bayesian optimization, we can more efficiently and effectively determine the optimal filter parameters, ultimately enhancing the performance of the denoising process while minimizing computational costs.

Bayesian optimization is advantageous due to its use of a probabilistic model to guide the search for optimal parameters. This approach can be illustrated using the concept of the acquisition function. The acquisition function helps determine where to sample next by balancing exploration (searching new areas) and exploitation (refining known good areas). A common acquisition function used in Bayesian optimization is the expected improvement (EI) function, which is defined as follows:

(1) $$EI\left(x\right)=E[\max\left(f\left(x^{\prime }\right)-f\left(x\right),0\right)]$$

where $f\left(x\right)$ is the objective function to be optimized, $x^{\prime }$ is the current best parameter setting, and $EI\left(x\right)$ represents the expected improvement of a new parameter setting *x* over the current best. This function measures the expected improvement in performance if a particular parameter setting *x* is chosen, guiding the optimization process to areas with high potential for improvement. In comparison to grid or random search methods, which lack a systematic approach to exploring parameter spaces, Bayesian optimization’s use of acquisition functions ensures more efficient and focused searches. This reduces the number of evaluations needed to find optimal parameters, making the process computationally more efficient and effective.

## 3. Methodology

In this section, we outline the framework and techniques employed in our approach to medical image denoising. We first explore the application of wavelet-based denoising as the foundational step, leveraging its strengths in decomposing the image into different frequency components. Following this, we incorporate a bilateral filter in the post-processing stage to further enhance the image quality by preserving edges while reducing noise. The optimization of the bilateral filter parameters is critical to achieving optimal results, and we employ Bayesian optimization for this purpose. Each component of our methodology is detailed in the subsequent sections.

### 3.1. Wavelet-Based Denoising

Wavelet-based denoising has emerged as a powerful and widely adopted technique in image processing, particularly for the task of image denoising. This method leverages the mathematical properties of wavelets, which allow for the decomposition of an image into different frequency components, enabling effective noise reduction while preserving important image details. In the context of image denoising, wavelets offer several advantages. Unlike traditional methods that may blur the edges and fine details of an image, wavelet-based techniques allow for multi-resolution analysis. This means the image is analyzed at various scales, making it possible to distinguish between noise and original image features at different levels of detail. The core idea behind wavelet-based denoising is to decompose the image into its wavelet coefficients using a wavelet transform. These coefficients represent the image’s details and approximations at different scales. Noise typically manifests as high-frequency components, which are more prominent in the finer scale coefficients. By applying thresholding techniques to the wavelet coefficients, these high-frequency noise components can be selectively reduced. After thresholding, the modified wavelet coefficients are used to reconstruct the denoised image through an inverse wavelet transform. The result is an image where noise has been effectively suppressed, yet the important structures and features of the original image remain largely intact. Using wavelet-based denoising for images involves several key steps: wavelet decomposition, thresholding, and reconstruction.

In this study, we utilized the Daubechies wavelet (db2) basis at five levels for wavelet transformation, which was selected for its computational simplicity and effectiveness in capturing sharp changes in image features. For thresholding, we employed the BayesShrink method [13], which adaptively determines the threshold by estimating the noise variance in each wavelet sub-band. This approach effectively separates noise from informative signal components. Additionally, we applied soft thresholding, which gradually reduces coefficients below the threshold, leading to smoother image reconstruction and better preservation of fine details. This combination ensures that the denoising process effectively reduces noise while maintaining important structural information.

The first step involves applying the wavelet transform to the noisy image. Suppose $I\left(x,y\right)$ represents the original noisy image. The discrete wavelet transform (DWT) of $I\left(x,y\right)$ is expressed as follows:

(2) $$W_{\psi }\left(j,m,n\right)=\sum_{x}\sum_{y}I\left(x,y\right)\cdot \psi_{j,m,n}(x,y)$$

where $W_{\psi }\left(j,m,n\right)$ are the wavelet coefficients at scale *j* and position $\left(m,n\right)$ and $\psi_{j,m,n}(x,y)$ are the wavelet basis functions. After decomposition, thresholding is applied to the wavelet coefficients to remove noise. The idea is that the coefficients corresponding to noise tend to have smaller magnitudes, as noise is usually represented by high-frequency components, while those representing significant image features, such as edges and textures, have larger magnitudes. By selectively shrinking or zeroing out the small-magnitude coefficients (through hard or soft thresholding), the noise can be effectively reduced. After thresholding, the denoised image is reconstructed by performing the inverse discrete wavelet transform (IDWT) on the modified wavelet coefficients, restoring the image with reduced noise while preserving essential details.

### 3.2. Bilateral Filter

The bilateral filter is a non-linear, edge-preserving, and noise-reducing smoothing filter that can be applied as a post-processing step after wavelet-based denoising. The key advantage of the bilateral filter is its ability to smooth the image while preserving edges, which is critical for maintaining important structural details.

The bilateral filter works by combining information from both the spatial domain and the intensity domain. For a pixel located at $\left(x,y\right)$ in an image $I\left(x,y\right)$, the output of the bilateral filter $I_{B}\left(x,y\right)$ is given by

(3) $$I_{B}\left(x,y\right)=\frac{1}{W_{P}}\sum_{x^{\prime }}\sum_{y^{\prime }}I\left(x^{\prime },y^{\prime }\right)\cdot exp(-\frac{{\left(x-x^{\prime }\right)}^{2}+{\left(y-y^{\prime }\right)}^{2}}{2\sigma_{s}^{2}})\cdot \exp\left(-\frac{{\left(I\left(x,y\right)-I\left(x^{\prime },y^{\prime }\right)\right)}^{2}}{2\sigma_{r}^{2}}\right)$$

where $\left(x^{\prime },y^{\prime }\right)$ are the coordinates of the neighboring pixels around $\left(x,y\right)$, $\sigma_{s}$ controls the spatial spread, determining how much influence neighboring pixels have based on their distance from the pixel at $\left(x,y\right)$, $\sigma_{r}$ controls the range spread, determining how much influence neighboring pixels have based on the similarity of their intensity values to the pixel at $\left(x,y\right)$, and $W_{P}$ is a normalization factor.

(4) $$W_{P}=\sum_{x^{\prime }}\sum_{y^{\prime }}\;exp(-\frac{{\left(x-x^{\prime }\right)}^{2}+{\left(y-y^{\prime }\right)}^{2}}{2\sigma_{s}^{2}})\cdot \exp\left(-\frac{{\left(I\left(x,y\right)-I\left(x^{\prime },y^{\prime }\right)\right)}^{2}}{2\sigma_{r}^{2}}\right)$$

The spatial weighting term $exp(-\frac{{\left(x-x^{\prime }\right)}^{2}+{\left(y-y^{\prime }\right)}^{2}}{2\sigma_{s}^{2}})$ ensures that pixels closer in space to $\left(x,y\right)$ have more influence, while the intensity weighting term $\exp\left(-\frac{{\left(I\left(x,y\right)-I\left(x^{\prime },y^{\prime }\right)\right)}^{2}}{2\sigma_{r}^{2}}\right)$ ensures that pixels with similar intensity values to $I\left(x,y\right)$ have more influence. In the context of image denoising, after wavelet-based denoising, the bilateral filter further smooths the image by reducing residual noise or artifacts, especially around edges, without blurring important structures, thus enhancing the overall quality of the denoised image.

Finding the optimal parameters in the bilateral filter, specifically the spatial spread ${(\sigma }_{s})$ and range spread ${(\sigma }_{r})$, is crucial for achieving the desired balance between noise reduction and edge preservation. If these parameters are not well tuned, the filter might either oversmooth the image, blurring important details, or fail to sufficiently reduce noise. Therefore, determining the best values for $\sigma_{s}$ and $\sigma_{r}$ is essential for maximizing the performance of the bilateral filter in image denoising. To address this challenge, we propose using a Bayesian optimization approach to systematically find the optimal parameters. Bayesian optimization is a probabilistic model-based optimization technique that is particularly effective for optimizing functions that are expensive to evaluate, such as image quality metrics. Bayesian optimization intelligently explores parameter space, balancing the exploration of new areas with the exploitation of known promising regions.

### 3.3. Bayesian Optimization

Finding the optimal parameters in the bilateral filter is crucial for achieving high-quality denoising results. The parameters of the bilateral filter, such as the filter size d, which determines the diameter of the region around each pixel, color sigma ${(\sigma }_{color})$, and spatial sigma ${(\sigma }_{space})$, directly affect the balance between noise reduction and edge preservation. To automate and optimize the selection of these parameters, we employ a Bayesian optimization approach.

In our approach, the objective function is defined based on the Peak Signal-to-Noise Ratio (PSNR) between the original image and the denoised image produced by the bilateral filter. The PSNR is a widely used metric for assessing image quality, with higher values indicating better preservation of the original image features after denoising. Formally, the objective function can be expressed as follows:

(5) $$Objective\;function=-PSNR(I_{original}-I_{filtered})$$

where $I_{original}$ is the original (clean) image and $I_{filtered}$ is the denoised image obtained by applying the bilateral filter with specific parameter settings. The negative PSNR is minimized to effectively maximize the PSNR. Bayesian optimization is particularly well suited for optimizing functions that are expensive to evaluate and where we have no closed-form expression for the objective function. It works by constructing an approximate model of the objective function, which is then used to predict the most promising parameter values to evaluate next.

**Figure 1:**
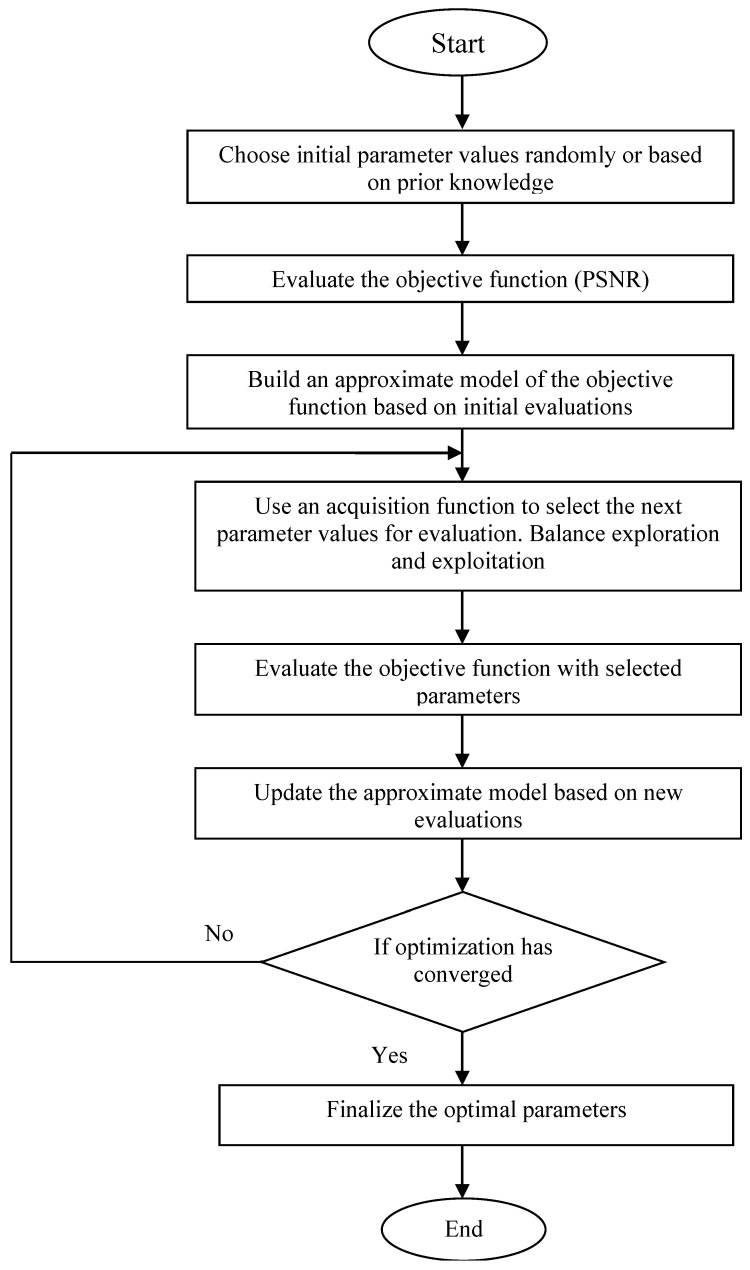
Flowchart of the Bayesian optimization process.

The Bayesian optimization process begins with the initialization step, where an initial set of parameter values is chosen, typically either randomly or based on prior knowledge. These initial parameters are then used to evaluate the objective function, which in this case is the PSNR. Following this, a model construction step occurs, where an approximate model of the objective function is built based on the initial evaluations. This model is designed to predict the PSNR for different combinations of the filter parameters. Next, the algorithm employs an acquisition function to decide on the next set of parameters to evaluate. The acquisition function balances the exploration of new parameter values and the exploitation of known good ones, guiding the selection process effectively. In the iteration step, the selected parameters are used to denoise the image, and the PSNR is calculated to evaluate performance. The approximate model is updated with each new evaluation, refining its predictions. This iterative process continues, with the acquisition function selecting new parameters based on the updated model, until the convergence criterion is met. Convergence is achieved when the algorithm identifies a set of parameters that maximizes the PSNR, indicating that the bilateral filter has been optimally tuned for the best possible denoising performance. If convergence is not achieved, the process returns to the acquisition function step to select a new set of parameters and continue the optimization. We defined a range of values for parameters such as d, color sigma ${(\sigma }_{color})$, and spatial sigma ${(\sigma }_{space})$, which represent our prior densities based on initial assumptions. Through the optimization process, we evaluated these parameters using performance metrics, leading to the identification of optimal values. The posterior densities reflect our updated understanding after considering the data. The final results are presented in Table 1 for Gaussian noise and Table 2 for Poisson noise. The flowchart of the Bayesian optimization process is depicted in Figure 1.

## 4. Experimental Results

In this section, we present the results of our proposed denoising method, which combines wavelet-based denoising with an optimized bilateral filter for medical images. The experiments were conducted on various medical image datasets to evaluate the method’s effectiveness in reducing noise while preserving essential image features. We demonstrate how our approach improves the visual quality and clarity of the images compared to traditional denoising techniques. Through a series of experiments, we highlight the benefits of using Bayesian optimization to fine tune the bilateral filter parameters, ensuring optimal performance in noise reduction and detail preservation.

The experimental evaluation was conducted using the Breast Ultrasound Images Dataset, which serves as a reliable basis for evaluating the effectiveness of the proposed methods [25]. This dataset features a diverse range of ultrasound imaging data collected from 600 female participants aged 25 to 75 years in 2018. It includes a total of 780 ultrasound images, each saved in PNG format with an average size of 500 × 500 pixels. The images are classified into three categories, normal, benign, and malignant, offering valuable insights into various breast health conditions. Specifically, the dataset contains 487 benign images, 210 malignant images, and 133 normal images, providing a comprehensive resource for analyzing breast tissue characteristics across different clinical scenarios [25].

In our experiments, we evaluated the performance of the proposed denoising method using two types of noise commonly encountered in medical imaging, Gaussian and Poisson noise. Gaussian Noise is statistical noise that follows a normal distribution and is characterized by its mean and standard deviation. In this study, Gaussian noise was configured with a mean value of 0 and a standard deviation of 25. The mean value of 0 indicates that the noise is centered on zero. The standard deviation of 25 determines the spread of the noise around this mean. Poisson noise arises from the inherent randomness in the number of photons detected by imaging sensors. The characteristics of Poisson noise are dependent on the signal intensity itself. In our experiments, the noise parameters were derived from the normalized pixel values of the image, which established the mean for the Poisson distribution at each pixel. This means that for each pixel, the noise follows a Poisson distribution with a mean equal to the pixel’s intensity value. By applying these noise models, we assessed the robustness of our proposed method under different noise conditions.

To comprehensively evaluate the performance of our proposed denoising method, we used several standard metrics: the Peak Signal-to-Noise Ratio (PSNR), the Mean Squared Error (MSE), and the Structural Similarity Index (SSIM). Each of these metrics provides unique insights into different aspects of image quality and denoising effectiveness.

- Peak Signal-to-Noise Ratio (PSNR).

The PSNR is a widely used metric that measures the quality of a denoised image compared to the original image. It is defined as follows:

(6) $$PSNR=10\;{log}_{10}(\frac{{\;\;\;MAX}^{2}}{MSE})$$

where MAX is the maximum possible pixel value of the image (255 for 8-bit images) and MSE is the Mean Squared Error. The PSNR is expressed in decibels (dB), with higher values indicating better image quality and less distortion. The PSNR quantifies the ratio of the maximum signal power to the noise power, making it a useful metric for evaluating how well the denoising algorithm preserves the original image details.

- Mean Squared Error (MSE).

The MSE measures the average squared differences between the original image and the denoised image. It is calculated as follows:

(7) $$MSE=\frac{1}{N}\sum_{i=1}^{N}{\left(I_{i}-{\hat{I}}_{i}\right)}^{2}$$

where $I_{i}$ represents the pixel value of the original image, ${\hat{I}}_{i}$ represents the pixel value of the denoised image, and *N* is the total number of pixels in the image. The MSE provides a quantitative measure of the difference between the original and denoised images, with lower values indicating better denoising performance and closer approximation to the original image.

- Structural Similarity Index (SSIM).

The SSIM is a metric that assesses the perceived quality of an image by evaluating structural information, luminance, and contrast. Unlike the PSNR and MSE, which primarily focus on pixel-wise differences, the SSIM considers changes in structural information that affect human visual perception. The SSIM is calculated using the following formula:

(8) $$SSIM\left(x,y\right)=\frac{\left(2{µ}_{x}{µ}_{y}+C_{1})(2\sigma_{xy}+C_{2}\right)}{\left({µ}_{x}^{2}+{µ}_{y}^{2}+C_{1})(\sigma_{x}^{2}+\sigma_{y}^{2}+C_{2}\right)}$$

where ${µ}_{x}$ and ${µ}_{y}$ are the mean pixel values, $\sigma_{x}^{2}$ and $\sigma_{y}^{2}$ are the variances, and $\sigma_{xy}$ is the covariance between *x* and *y*. $C_{1}$ and $C_{2}$ are constants to stabilize the division. SSIM values range from −1 to 1, with a value of 1 indicating perfect structural similarity between the original and denoised images.

In this section, we present the findings from our experiments. First, we discuss the results of the Bayesian optimization process used to fine tune the parameters of the bilateral filter. Then, we evaluate the effectiveness of the optimized denoising method by analyzing the performance metrics obtained for images denoised under different noise conditions.

### 4.1. Results of Bayesian Optimization

To optimize the parameters of the bilateral filter for effective denoising, we employed Bayesian optimization. The primary objective was to maximize the PSNR of the denoised images compared to the original, noise-free images. The Bayesian optimization process iteratively evaluated different parameter combinations for the bilateral filter, specifically the filter size (*d*), sigma color ${(\sigma }_{color})$, and sigma space ${(\sigma }_{space})$. The goal was to find the parameter set that provides the best balance between noise reduction and detail preservation.

After running the optimization for 100 iterations, the best parameter values were identified. These parameters significantly enhanced the denoising performance, yielding a higher PSNR value, which indicates better image quality. The optimization process efficiently explored the parameter space, focusing on the optimal configuration that provided substantial noise reduction while maintaining the essential structural details of the images. The results of the Bayesian optimization, detailing the best parameters for denoising images corrupted with Gaussian noise and Poisson noise, are summarized in Table 1 and Table 2, respectively.The local identifiers used in this study correspond to the following images from the dataset, Images I, II, III, IV, and V, refer to the normal images (1, 2, 3, 4, and 5), while Images VI, VII, and VIII correspond to benign images (437, 10, and 20), and Images IX and X represent malignant images (204 and 210). 

**Table 1:** Optimized bilateral filter parameters for Gaussian noise.

Gaussian	Image I	Image II	Image III	Image IV	Image V	Image VI	Image VII	Image VIII	Image IX	Image X
*d*	7	7	7	6	6	6	6	6	7	7
$\sigma_{color}$	140	135	135	140	140	140	140	136	140	140
$\sigma_{space}$	10	10	10	10	10	10	10	10	10	10

**Table 2:** Optimized bilateral filter parameters for Poisson noise.

Poisson	Image I	Image II	Image III	Image IV	Image V	Image VI	Image VII	Image VIII	Image IX	Image X
*d*	4	4	4	4	5	4	4	4	4	4
$\sigma_{color}$	110	86	97	95	140	134	123	100	124	132
$\sigma_{space}$	10	10	10	10	10	10	10	10	10	10

### 4.2. Denoising Performance Analysis

To evaluate the effectiveness of our proposed denoising method under Gaussian noise, we compared its performance against several well-known denoising techniques: the median filter, non-local means (NLM), bilateral filter, and wavelet-based denoising. We used three standard evaluation metrics, the Peak Signal-to-Noise Ratio (PSNR), the Mean Squared Error (MSE), and the Structural Similarity Index (SSIM), to assess the quality of the denoised images. These metrics provide a comprehensive view of both the fidelity of the denoised images (through PSNR and MSE) and the preservation of structural information (through SSIM). To evaluate performance, we selected a representative sample of images to demonstrate the effectiveness of the proposed method. The results from this subset align closely with the performance observed across the entire dataset, indicating the robustness of our approach. By showcasing these example images, we provide a clear illustration of the denoising capabilities without compromising the scope of our analysis.

The experimental results indicate that our proposed method outperforms the traditional approaches in terms of higher PSNR values, lower MSE values, and higher SSIM scores, demonstrating improved noise reduction while preserving essential image details. This improvement can be attributed to the optimized bilateral filter parameters, which effectively balance noise removal and detail preservation. Specifically, our method shows significant enhancement in image clarity and structural similarity compared to the median filter, non-local means, and traditional bilateral and wavelet-based methods. The comparative performance of the proposed method and the other denoising techniques under Gaussian noise is summarized in Table 3. This table illustrates the effectiveness of our approach in maintaining image quality while minimizing noise, validating its effectiveness for medical image denoising.

To further highlight the effectiveness of our proposed denoising method, we calculated the percentage improvement in the PSNR, MSE, and SSIM metrics compared to traditional denoising techniques, median filter, non-local means (NLM), bilateral filter, and wavelet-based denoising under Gaussian noise conditions. The percentage improvement provides a quantitative measure of how much better our method performs relative to these widely used techniques. The results, depicted in Table 4, show that our proposed method achieves substantial improvements in the PSNR, indicating a higher level of noise suppression while maintaining image quality. Similarly, the lower MSE values reflect more accurate pixel value restoration, and the higher SSIM scores confirm better preservation of structural information. The significant percentage gains across all three metrics underscore the enhanced performance of our method, validating its effectiveness as a robust denoising approach for medical images affected by Gaussian noise.

In scenarios where the original image is unavailable, the practical applicability of the proposed method can still be demonstrated. After obtaining the optimal parameters for denoising based on one reference image, these parameters are applied across all images in the dataset. This approach allows for consistent performance without relying on the original image for comparison, making it adaptable to real-world situations where such ground truth references are typically not present. By utilizing this strategy, the method ensures practicality in applications beyond controlled experimental settings. Based on the optimal parameters determined from the reference image, with d = 7, sigma color = 140, and sigma space = 10, these values were applied to all images in the dataset. The results, as shown in Table 5, demonstrate that the proposed method performs effectively across the dataset. When comparing the outcomes in Table 3 and Table 5, there are no significant differences, indicating the robustness of the proposed method.

**Figure 2:**
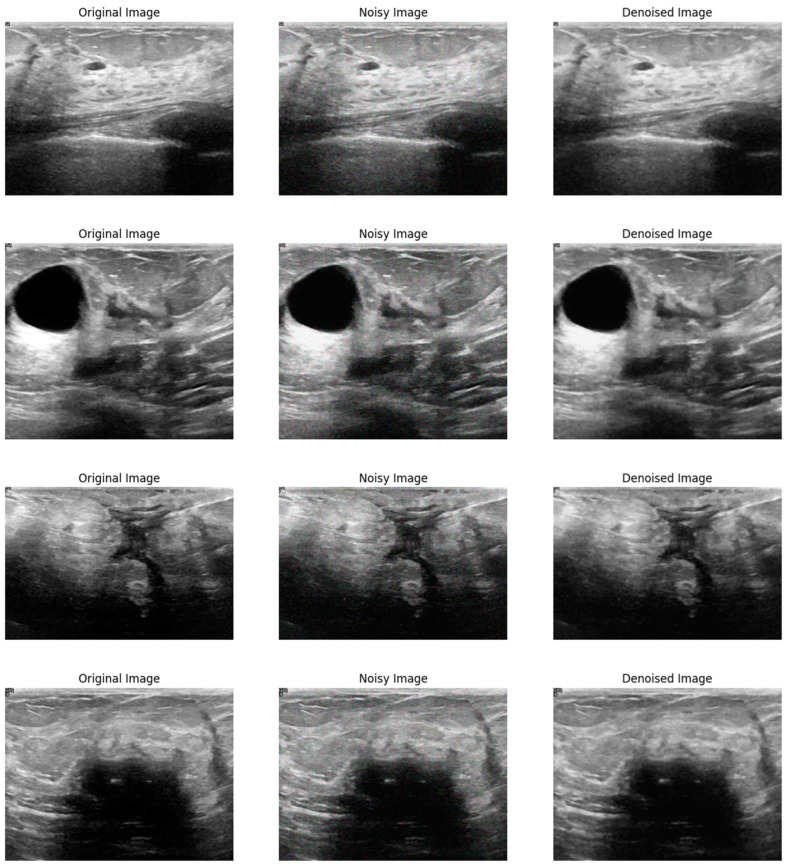
Visual comparison of the proposed method on Gaussian noise.

Figure 2 illustrates the visual effectiveness of the proposed denoising method. The figure displays three images for comparison: the original noise-free image, the image corrupted by Gaussian noise, and the image after being denoised using the proposed method. This visual comparison provides a qualitative assessment of the denoising performance, supplementing the quantitative results discussed earlier. The original image serves as a reference, showing the image without any noise interference. The noisy image, corrupted by Gaussian noise, exhibits significant visual distortions that obscure finer details and compromise the visibility of important structural information. In contrast, the denoised image produced by the proposed method shows a remarkable reduction in noise levels, with enhanced clarity and preservation of structural details. This comparison clearly demonstrates the ability of the proposed method to effectively remove noise while maintaining the quality and integrity of the original image.

To evaluate the robustness of our proposed denoising method in different noise scenarios, we compared its performance under Poisson noise with several widely used denoising techniques: median filter, non-local means (NLM), bilateral filter, and wavelet-based denoising. The results, presented in Table 6, demonstrate that the proposed method consistently outperforms the traditional denoising techniques. Specifically, our method achieves higher PSNR values, indicating better noise suppression and enhanced image clarity. It also results in lower MSE values, reflecting more accurate restoration of pixel intensities, and higher SSIM scores, showing improved preservation of structural information compared to the other methods. These findings highlight the effectiveness of the proposed approach in maintaining image quality while effectively handling the noise characteristics typical of Poisson noise.

To quantify the relative performance gains of our proposed method compared to the traditional denoising techniques under Poisson noise, we calculated the percentage improvement in the PSNR, MSE, and SSIM metrics. This analysis highlights how much better our method performs in terms of noise reduction and image quality preservation compared to the median filter, NLM, bilateral filter, and wavelet-based denoising techniques. The percentage improvements, detailed in Table 7, show the relative enhancements achieved by the proposed method over these other techniques. By presenting these improvements, we provide a clear measure of how effectively our method surpasses the conventional approaches in handling Poisson noise. The results demonstrate significant gains in the PSNR, indicating better noise suppression, a lower MSE, reflecting more accurate pixel recovery, and a higher SSIM, showing better structural preservation.

When the original image is not available in practical scenarios, the optimal parameters are determined from a reference image and then applied across all images in the dataset. For Poisson noise, the best values were found to be d = 4, sigma color = 110, and sigma space = 10. The results, as shown in Table 8, indicate consistent performance across the dataset. Moreover, a comparison of the results between Table 6 and Table 8 reveals no significant differences, underscoring the robustness and effectiveness of the proposed method in various contexts.

**Figure 3:**
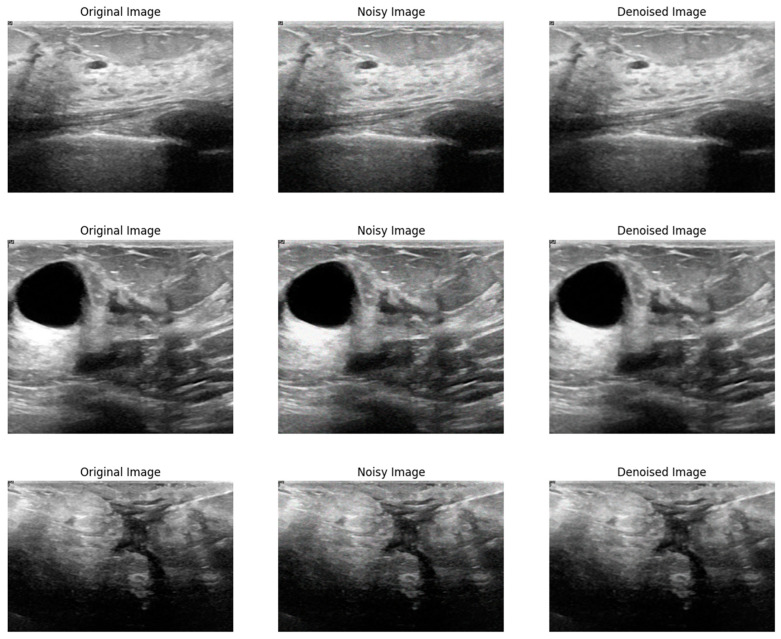

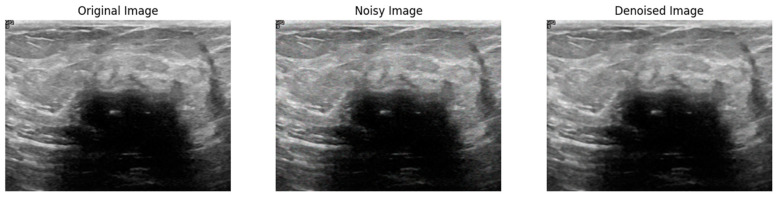
Visual comparison of the proposed method on Poisson noise.

Figure 3 shows a visual comparison of the original image, the image corrupted by Poisson noise, and the denoised image produced using the proposed method. The original image serves as a baseline, displaying clear and noise-free content. The second image illustrates the significant noise introduced by Poisson distortion, which impacts image clarity and detail. In contrast, the final image demonstrates the effectiveness of the proposed denoising method, showing a substantial reduction in noise and an enhancement in image quality while preserving critical details. This comparison highlights the ability of the proposed method to effectively restore the image and mitigate the adverse effects of Poisson noise.

## 5. Conclusions

In this study, we have developed an advanced approach to medical image denoising by integrating wavelet-based techniques with bilateral filtering. Our wavelet method effectively reduces noise while preserving essential image details, and the subsequent application of bilateral filtering further enhances image quality by maintaining edge sharpness. The key to optimizing the bilateral filter’s performance was the careful tuning of its parameters, achieved through Bayesian optimization. This technique provided a systematic and efficient way to find the optimal settings, leading to significant improvements in both noise reduction and detail preservation. The experimental results confirm that our combined approach offers a robust solution for enhancing medical images, demonstrating substantial gains over conventional methods.

## Figures and Tables

**Table 3:** Performance comparison of denoising methods under Gaussian noise.

Gaussian Noise		Image I	Image II	Image III	Image IV	Image V	Image VI	Image VII	Image VIII	Image IX	Image X
Median	PSNR	27.62	27.34	27.54	27.45	27.69	27.73	27.78	27.62	28.02	27.87
	MSE	0.0017	0.0018	0.0017	0.0017	0.0017	0.0016	0.0016	0.0017	0.0015	0.0016
	SSIM	0.491	0.529	0.520	0.508	0.504	0.495	0.514	0.480	0.492	0.499
NLM	PSNR	24.20	23.50	23.24	24.95	25.96	25.17	25.32	23.20	24.07	25.21
	MSE	0.0038	0.0044	0.0047	0.0031	0.0025	0.0030	0.0029	0.0047	0.0039	0.0030
	SSIM	0.458	0.434	0.390	0.526	0.610	0.568	0.553	0.367	0.432	0.532
Bilateral	PSNR	29.70	28.31	28.82	28.77	29.28	29.59	29.04	30.06	29.92	29.53
	MSE	0.0010	0.0014	0.0013	0.0013	0.0011	0.0011	0.0012	0.0009	0.0010	0.0011
	SSIM	0.659	0.643	0.670	0.628	0.633	0.645	0.619	0.682	0.642	0.629
Wavelet	PSNR	29.86	28.43	28.78	28.97	29.77	30.10	29.21	29.80	30.16	29.73
	MSE	0.0010	0.0014	0.0013	0.0013	0.0011	0.0010	0.0012	0.0010	0.0010	0.0011
	SSIM	0.689	0.640	0.665	0.622	0.681	0.692	0.644	0.689	0.672	0.658
Proposed Method	PSNR	33.45	31.51	31.89	31.92	33.76	33.77	32.69	32.32	33.07	32.84
	MSE	0.0005	0.0007	0.0006	0.0006	0.0004	0.0004	0.0005	0.0006	0.0005	0.0005
	SSIM	0.867	0.832	0.852	0.816	0.885	0.883	0.839	0.837	0.838	0.834

**Table 4:** Percentage improvement of performance metrics of the proposed method under Gaussian noise.

Gaussian Noise	Median			NLM			Bilateral			Wavelet		
	PSNR	MSE	SSIM	PSNR	MSE	SSIM	PSNR	MSE	SSIM	PSNR	MSE	SSIM
Improvement %	18.26	68.14	68.73	33.77	84.99	78.56	11.67	53.11	31.61	10.99	53.42	27.59

**Table 5:** Denoising performance using optimized parameters across the dataset under Gaussian noise.

Gaussian Noise		Image I	Image II	Image III	Image IV	Image V	Image VI	Image VII	Image VIII	Image IX	Image X
Proposed Method	PSNR	33.45	31.45	31.84	31.92	33.76	33.77	32.69	32.29	33.07	32.84
	MSE	0.0005	0.0007	0.0007	0.0006	0.0004	0.0004	0.0005	0.0006	0.0005	0.0005
	SSIM	0.867	0.831	0.852	0.816	0.885	0.883	0.839	0.836	0.837	0.834

**Table 6:** Performance comparison of denoising methods under Poisson noise.

Poisson Noise		Image I	Image II	Image III	Image IV	Image V	Image VI	Image VII	Image VIII	Image IX	Image X
Median	PSNR	32.92	32.32	32.83	31.74	32.11	32.15	32.35	32.92	33.48	32.59
	MSE	0.0005	0.0005	0.0005	0.0006	0.0006	0.0006	0.0005	0.0005	0.0004	0.0005
	SSIM	0.790	0.804	0.825	0.747	0.750	0.754	0.772	0.801	0.802	0.780
NLM	PSNR	32.73	31.97	32.52	30.88	32.11	32.01	32.17	32.24	33.55	32.70
	MSE	0.0005	0.0006	0.0005	0.0008	0.0006	0.0006	0.0006	0.0005	0.0004	0.0005
	SSIM	0.878	0.868	0.887	0.831	0.880	0.878	0.870	0.857	0.893	0.879
Bilateral	PSNR	32.08	30.29	30.88	30.85	31.50	31.71	31.45	32.22	32.41	31.99
	MSE	0.0006	0.0009	0.0008	0.0008	0.0007	0.0006	0.0007	0.0006	0.0005	0.0006
	SSIM	0.852	0.825	0.839	0.823	0.837	0.834	0.839	0.841	0.838	0.832
Wavelet	PSNR	31.69	31.16	30.57	31.47	31.75	31.03	31.54	30.61	30.69	30.63
	MSE	0.0007	0.0008	0.0009	0.0007	0.0007	0.0008	0.0007	0.0009	0.0009	0.0009
	SSIM	0.753	0.761	0.757	0.742	0.741	0.711	0.750	0.743	0.705	0.695
Proposed Method	PSNR	36.85	35.55	35.86	34.98	36.85	36.79	36.35	35.55	37.46	36.88
	MSE	0.0002	0.0003	0.0003	0.0003	0.0002	0.0002	0.0002	0.0003	0.0002	0.0002
	SSIM	0.918	0.911	0.920	0.887	0.925	0.920	0.921	0.897	0.919	0.915

**Table 7:** Percentage improvement of performance metrics of the proposed method under Poisson noise.

Poisson Noise	Median			NLM			Bilateral			Wavelet		
	PSNR	MSE	SSIM	PSNR	MSE	SSIM	PSNR	MSE	SSIM	PSNR	MSE	SSIM
Improvement %	11.59	53.33	16.82	12.47	56.25	4.74	15.15	64.45	9.25	16.73	69.78	24.24

**Table 8:** Denoising performance using optimized parameters across the dataset under Poisson noise.

Poisson Noise		Image I	Image II	Image III	Image IV	Image V	Image VI	Image VII	Image VIII	Image IX	Image X
Proposed Method	PSNR	36.85	35.30	35.77	34.88	36.93	36.83	36.39	35.49	37.48	36.93
	MSE	0.0002	0.0003	0.0003	0.0003	0.0002	0.0002	0.0002	0.0003	0.0002	0.0002
	SSIM	0.918	0.908	0.919	0.885	0.925	0.920	0.921	0.896	0.919	0.916

## Data Availability

The data can be shared up on request.
